# Multidisciplinary treatment based on surgery leading to long-term survival of a patient with multiple asynchronous rare primary malignant neoplasms: A case report and literature review

**DOI:** 10.3892/ol.2014.2833

**Published:** 2014-12-29

**Authors:** HONG-LIN GU, SHI-XING ZENG, YUN-BING CHANG, ZHEN LIN, QIU-JIAN ZHENG, XIAO-QING ZHENG, ZHEN-WEI PENG, SHI-QIANG ZHAN

**Affiliations:** 1 Department of Othopedics, Guangdong Academy of Medical Science, Guangdong General Hospital, Guangdong 510080, P.R. China; 2 Department of Oncology, The First Affiliated Hospital, Sun Yat-sen University, Guangzhou, Guangdong 510080, P.R. China

**Keywords:** multiple primary malignant neoplasms, epithelioid hemangioendothelioma, Ewing’s sarcoma, malignant solitary fibrous tumour, multidisciplinary treatment

## Abstract

Patients that present with multiple primary malignant neoplasms are increasingly encountered, but the treatment of such patients presents specific challenges and long-term survival is rare. The present study reports the case of a 45-year-old female diagnosed with three rare, distinct primary malignant neoplasms, including epithelioid hemangioendothelioma (EHE) of the brain, Ewing’s sarcoma of the lumbar 2 vertebra and a malignant solitary fibrous tumour (SFT) of the liver, at different time points. The patient underwent multidisciplinary treatment according to the diagnoses, including radial resection of all primary lesions, chemotherapy (consisting of vincristine, dactinomycin, cyclophosphamide and adriamycin) and radiotherapy, to treat Ewing’s sarcoma and metastases of EHE and malignant SFT. Following these treatments, the patient survived for >14 years. Multidisciplinary treatment regimens based on surgery can lead to long-term survival of patients with multiple asynchronous rare primary malignant neoplasms. The present study reported that multidisciplinary treatment regimens based on surgery can lead to the long-term survival of patients with multiple asynchronous rare primary malignant neoplasms.

## Introduction

The occurrence of multiple primary malignant neoplasms (MPMNs) is increasing, with the reported incidence ranging between 0.734 and 11.3% in India (1). In patients with neoplastic disease, an early diagnosis and treatment with chemo- and radiotherapy improves long-term survival, thus increasing the risk of developing subsequent primary tumours (2). Furthermore, with improvements in diagnostic modalities, including positron emission tomography (PET), indolent tumour detection rates have increased, contributing to the apparent increase in the incidence of multiple primary malignancies (3).

Studies on the survival of patients with MPMNs are limited, possibly due to the particularly heterogeneous nature of this group (4). It has been suggested that the presence of multiple cancers does not affect the overall survival rate of patients with MPMNs (5), and that the overall survival rate is similar to that of patients with single lesion tumour types (6). However, according to the literature, patients with three asynchronous primary malignant neoplasms exhibit an extremely poor prognosis (7,8).

In the current study, a patient presented with three rare distinct primary malignant neoplasms, consisting of epithelioid hemangioendothelioma (EHE) of the brain, Ewing’s sarcoma of the lumbar vertebrae and malignant solitary fibrous tumour (SFT) of the liver. The primary Ewing’s sarcoma affected the lumbar 2 (L2) vertebra, as did metastases of the EHE and malignant SFT. The patient received multidisciplinary treatment and survived for >14 years. Written informed consent was obtained from the patient’s family.

## Case report

A 31-year-old female presented with severe headache upon wakening for 6 months and was referred to The First Affiliated Hospital of Sun Yat-sen University (Guangzhou, China) in May 1999. No other symptoms were noted during a physical examination. Head computed tomography (CT) scans revealed a slightly hyperintense mass in the left parietal region. Radionuclide bone scanning, chest radiography and abdominal ultrasonography revealed no evidence of malignant lesions in other organs. The patient underwent surgical excision of the left parietal lesion. Histopathological analysis of the resected tumour revealed an EHE with a cluster of differentiation (CD)34-positive and factor VIII-associated antigen-positive immunophenotype. A post-operative follow-up, which lasted for five years, revealed no evidence of recurrence.

**Figure 1:**
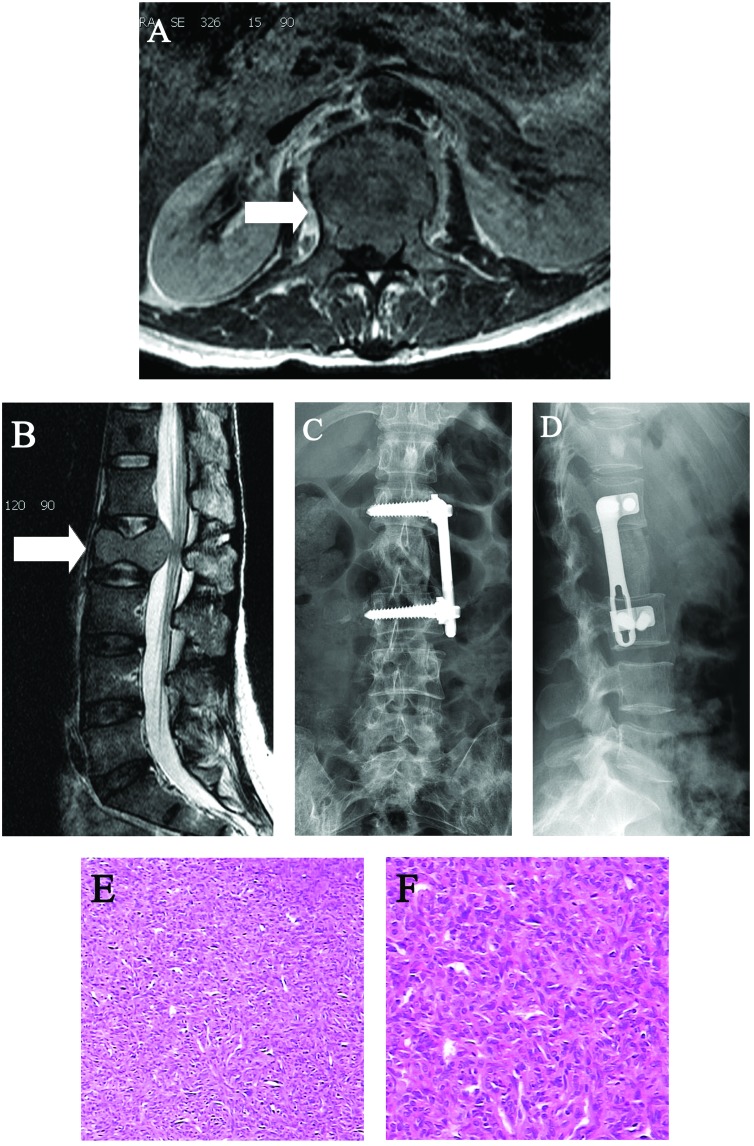
Pre- and post-operative radiographic images and pathological appearance of the L2 metastatic EHE. (A and B) Pre-operative magnetic resonance imaging scan revealing a compression fracture and a slightly hyperintense mass on the L2 vertebra with compression of the spinal cord (arrows). (C and D) Post-operative radiograph subsequent to anterior L2 vertebrectomy and L1–L3 fusion. (E and F) Post-operative histopathology revealed the tumour to be EHE that was composed of vascular channels lined by atypical cells with an epithelioid shape and clear cytoplasm (magnification, ×100 and ×200, respectively; stain, hematoxylin and eosin). L, lumbar; EHE, epithelioid hemangioendothelioma.

In November 2004, the patient reported lower back and left leg pain. A magnetic resonance imaging (MRI) scan revealed a compression fracture and slightly hyperintense mass in the L2 vertebra, as well as compression of the spinal cord (Fig. 1A and B). Following the exclusion of other organ involvement by radionuclide bone scanning, chest radiography and abdominal ultrasonography, an anterior L2 vertebrectomy and L1–L3 fusion were performed (Fig. 1C and D). The post-operative histopathological examination indicated that the lesion was metastatic EHE (Fig. 1E and F). The patient was administered with radiotherapy at a dose of 44 Gy for 22 cycles. The patient did not experience any adverse reactions in response to the radiotherapy.

**Figure 2:**
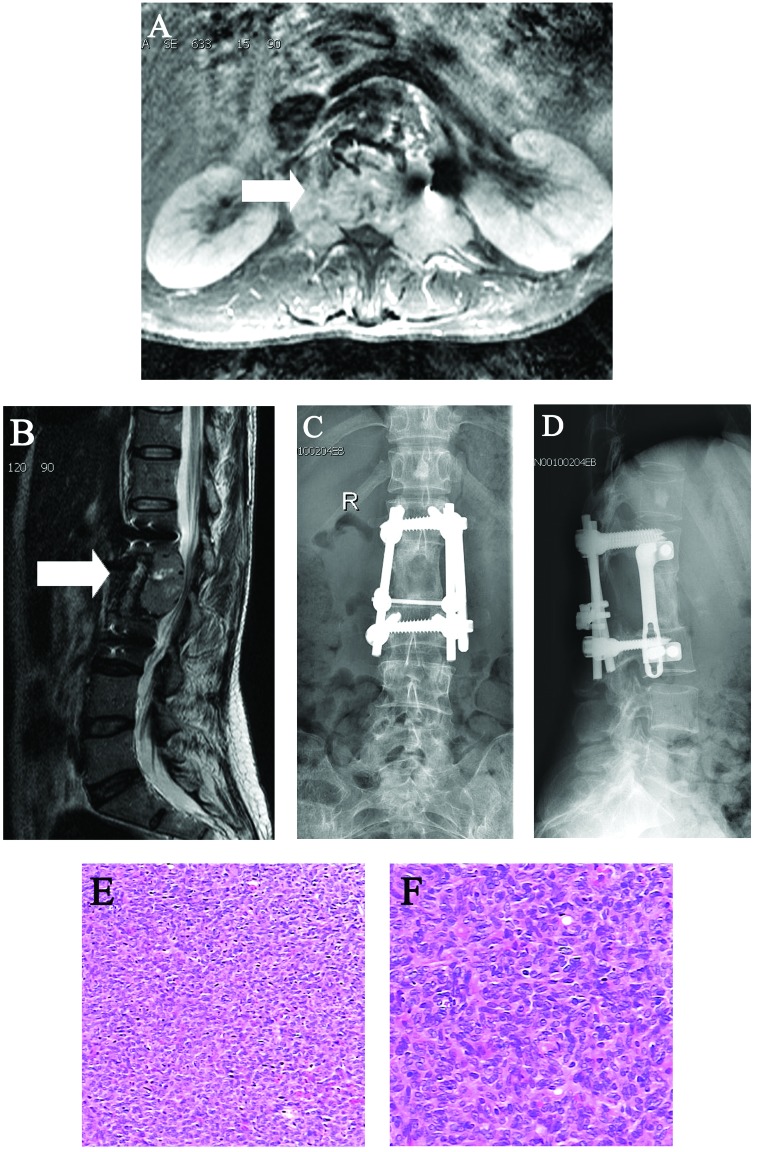
Pre- and post-operative radiographic images and pathological appearance of the L2 primary Ewing’s sarcoma. (A and B) Pre-operative magnetic resonance imaging revealing a posterior enhancing mass and spinal cord compression at the L2 vertebrae level with involvement of the bilateral vertebral lamina and superior processus (arrows). (C and D) Post-operative radiograph subsequent to posterior lesion excision and pedicle screw fixation. (E and F) Post-operative histopathology denoting Ewing’s sarcoma, composed of small blue tumour cells (magnification, ×100 and ×200, respectively; stain, hematoxylin and eosin). L2, lumbar 2.

In April 2007, the patient presented with recurrent lower back and left leg pain. An MRI scan revealed a posterior enhancing mass and spinal cord compression at the L2 vertebra level, with involvement of the bilateral vertebral lamina and superior processes (Fig. 2A and B). The involvement of other organs was excluded. Following a pre-operative diagnosis of recurrent EHE, a posterior lesion excision was performed with pedicle screw fixation (Fig. 2C and D). However, the subsequent histopathological examination indicated primary lumbar Ewing’s sarcoma with a vimentin-positive and microneme protein 2 (MIC2)-positive immunophenotype (Fig. 2E and F), for which the patient received 22 cycles of radiotherapy at a dose of 40 Gy and six cycles of chemotherapy, consisting of 2 mg vincristine, 0.7 mg dactinomycin, 700 mg cyclophosphamide and 40 mg adriamycin, over an 18-week period. The patient experienced mild gastrointestinal symptoms and impaired liver function during the treatment phase.

**Figure 3:**
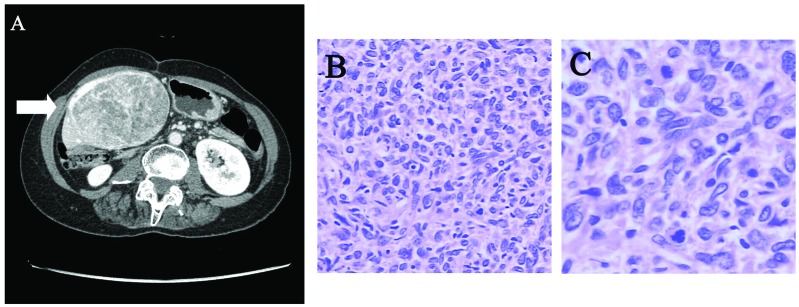
Pre-operative radiographic image and pathological appearance of the hepatic primary malignant SFT. (A) An abdominal computed tomography scan revealing a hyperintense mass in the S5 hepatic segment with a maximum diameter of 10 cm (arrow). (B and C) Post-operative histopathology revealed a malignant SFT composed of oval or round cells with cytoplasmic hyaline grains and a hemangiopericytoma-like vascular pattern (maginfication, ×100 and ×200, respectively; stain, hematoxylin and eosin). SFT, solitary fibrous tumour.

In May 2009, the patient presented with an abdominal mass. An abdominal CT scan revealed a hyperintense mass in the S5 hepatic segment, with a maximum diameter of 10 cm (Fig. 3A). The involvement of other organs was excluded. Hepatic segmentectomy was conducted to remove the S5 hepatic segment and the tumour. The histopathological report indicated a primary malignant SFT (Fig. 3B and C) with a CD34-positive and B-cell lymphoma (Bcl)-2-positive immunophenotype.

**Figure 4:**
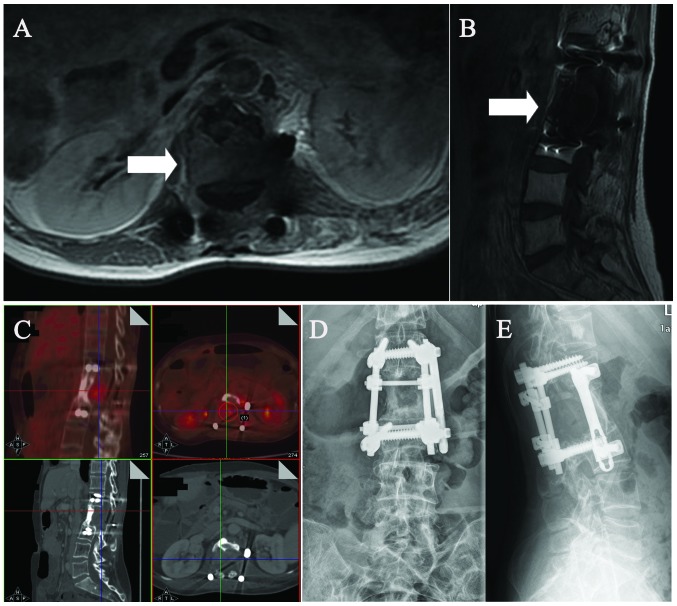
Pre- and post-operative radiographic images of the L2 metastatic malignant solitary fibrous tumour. (A and B) Magnetic resonance imaging scan revealing a posterior enhancing mass and spinal cord compression at the L2 level (arrows). (C) Positron emission tomography-computed tomography scan revealing a focal high uptake in the L2 level. (D and E) Post-operative radiograph subsequent to posterior palliative resection of the lumbar mass and re-fixation of larger pedicle screws. L2, lumbar 2.

In March 2010, the patient presented again with recurrent lower back and left leg pain. MRI and PET-CT scans revealed a posterior enhancing mass and spinal cord compression at the L2 vertebral level (Fig. 4A–C). Recurrent lumbar Ewing’s sarcoma was diagnosed and, subsequent to discussion with the patient, a palliative resection of the lumbar mass was performed to decompress the spinal cord (Fig. 4D and E). The post-operative histopathological examination indicated that the lesion was metastatic malignant SFT and palliative chemoradiotherapy was therefore initiated.

In April 2013, the patient experienced fever, dyspnoea and bone pain throughout the body. Radionuclide bone scanning and chest radiography revealed multiple bone metastases and a pulmonary infection. Laboratory examinations revealed pancytopenia. The patient was treated with palliative care and symptomatic therapy, but succumbed to respiratory-circulatory failure in June 2013.

## Discussion

Multiple primary neoplasms in a single patient were first reported at the end of the 19th century (9). The criteria for the definition of multiple primary neoplasms was provided by Warren and Gates in 1932 (10), who specified that each tumour must be distinct and present a definitive pattern of malignant disease, and that the possibility of metastasis from a primary tumour must be excluded. Tumours were defined as synchronous if occurring within six months of the diagnosis of the cancer and asynchronous if occurring more than six months prior to or following the diagnosis. In the present case, the patient was diagnosed with three rare distinct primary tumours, thus meeting the criteria for a diagnosis of triple asynchronous PMNs. MPMNs with three distinct lesions are considered extremely rare (11). MPMNs most commonly occur in the respiratory, gastrointestinal and genitourinary systems (10), and the involvement of brain, bone and liver tissues has rarely been reported (4). In addition, the incidence of EHE of the brain, Ewing’s sarcoma of the lumbar vertebra and malignant SFT of the liver, respectively, is extremely low, making the current case unique. EHE is an extremely rare intracranial tumour (12) that frequently affects the soft tissues, lung, liver and bone. The clinical manifestations of EHE depend on the location of occurrence, and the majority of patients present with a mass and symptoms of intracranial hypertension. Intracranial EHE lesions appear as isointense, hyperintense or heterogeneous masses on pre-operative MRI T1- and T2-weighted images (13), and vascular flow voids are observed on T2-weighted images (14). EHE tumours are detected by positive immunostaining for endothelial cell markers, including CD31, CD34 and factor VIII-associated antigen (15). In the present study, CD34 and factor VIII-associated antigen immunopositive tumour cells were detected, indicating the presence of EHE.

Ewing’s sarcoma of the bone most frequently involves the pelvic bones and femur (16), and although the vertebral column is frequently involved in metastatic disease (17), primary vertebral Ewing’s sarcoma is rare, with a reported incidence of 3.5–15% of all cases (18,19). The clinical manifestations of Ewing’s sarcoma that arise primarily in the spinal epidural space include back pain with or without radicular pain, paresis in one or two legs, sensory disturbances, and bladder and bowel dysfunction (20). The severe lower back pain and radicular leg pain experienced by the present patient were indicative of Ewing’s sarcoma. On MRI scans, Ewing’s sarcoma exhibits a low to isointense signal on T1-weighted images, high signal intensity on T2-weighted images and heterogeneous enhancement (21). Characteristic histological features of Ewing’s sarcoma include narrow sheets of poorly-differentiated cells exhibiting uniform round or oval nuclei and scant cytoplasm (22). Immunohistochemical analysis of Ewing’s sarcoma typically reveals the expression of vimentin and MIC2, as was observed in the present case.

Hepatic SFT is rare (23) and exhibits non-specific clinical manifestations. It can be asymptomatic and demonstrate increased abdominal volume, as was observed in the current case, or can present with symptoms that include nausea, vomiting, abdominal pain and hypoglycaemia (24,25). The majority of hepatic SFTs are benign and few studies reporting local recurrence or metastases exist (26). The current case is remarkable as lumbar metastases were apparent. The imaging features of SFT are also non-specific and cannot be used to distinguish between benign and malignant tumours. The lesion is usually a single large, well-circumscribed, heterogeneously enhancing mass on CT and MRI images (24). SFT cells are positive for CD34 in 90–95% of tumours, MIC2 in 70% of tumours and Bcl-2 in >80% of tumors (27). In the present case, the tumour cells were strongly positive for CD34 and Bcl-2, but were negative for CD117, smooth muscle antigen, S-100, EMA and desmin expression, indicating that the tumour was an SFT.

In the present study, the interval between the occurrences of the first two primary neoplasms was eight years, and the subsequent intervals were two years. Similar to other reported cases, the mean interval between the diagnoses of the first two primary tumours is always greater than the interval between the detections of subsequent neoplasms, the cause of which has yet to be elucidated (28). There are numerous possible reasons for the development of MPMNs, including prior chemo- or radiotherapy, lifestyle choices, such as tobacco, alcohol and diet, environmental exposures, host determinants, such as genetic predisposition or immune dysfunction, and combination effects, such as gene-environment and gene-gene interactions (29). Treatment-associated exposure to high doses of radiation or alkylating agents has been implicated in the development of subsequent malignancies (30). In the current case, the second and the third primary malignant neoplasms developed following chemoradiotherapy, which may have contributed to the development of MPMNs to a certain extent. A notable phenomenon was that the L2 vertebra was the sole vertebral site for the primary Ewing’s sarcoma, the metastatic EHE and the malignant SFT. Among patients with cancer, 12–20% present initially with spinal column metastases, 30% of which occurs in the lumbosacral region, but rarely in a single vertebra, indicating that the initial radiotherapy in this region may have been the cause of subsequent malignancies. In addition, the risk of multiple malignancies appears to be impacted by age at the time of diagnosis of the first tumour (31). A review of the literature determined that patients with MPMNs tend to be older than those with a single primary malignant neoplasm, with >75% of patients with MPMNs being >50 years old (32). However, the present patient was 45 years old at the time of writing the current study.

Treatment strategies for patients with MPMNs differ from the strategies used for patients with metastatic or recurrent cancer. Therefore, a novel tumour in a patient with cancer should be considered not only as recurrence or metastasis but also as a different primary malignancy. For asynchronous primary malignant neoplasms, it has been recommended for each tumour to be evaluated and staged as an independent tumour and treated aggressively with curative intent to achieve the maximum therapeutic benefit (1). If surgery is indicated for the two tumours, the procedure can be performed in a single-stage setting in the majority of cases, with low rates of morbidity and mortality (33). In the current case, the three primary malignant neoplasms were treated differently according to the individual tumour characteristics.

Surgical removal is the recommended treatment for EHE (34), and pre- and post-operative adjuvant therapies, consisting of chemo- or radiotherapy, are recommended for cases in which total resection is not possible, and those involving metastasis or multiple tumours (13,34). The initial excision of the intracranial EHE in the current case proved successful, with no complications or symptoms until the metastasis of EHE in the L2 vertebra five years later, which required chemoradiotherapy.

Current protocols for the treatment of Ewing’s sarcoma include radical excision, which generally exhibits a good prognosis (35), followed by radiotherapy and multidrug chemotherapy. In the current case, the Ewing’s sarcoma was pre-operatively diagnosed as recurrent EHE. Therefore, the patient underwent radical excision and chemoradiotherapy, with no evidence of recurrence being found during the follow-up.

The recommended treatment for hepatic SFT is radical removal of the tumour with clear margins of resection, with post-operative adjuvant therapy reserved for cases of incomplete resection or cases exhibiting histopathological features of malignancy (36). In the current case, radical resection was performed on the primary hepatic SFT without subsequent chemoradiotherapy in 2009. However, one year later, the lumbar metastasis of SFT was indentified and adjuvant chemoradiotherapy was initiated following surgical resection of the metastatic lesion.

Depending upon the site, pathological pattern, tumour variant, type of treatment and clinical series, the five-year survival rate of patients with MPMN can range between 33 and 65% (37,38). Gursel *et al* reported the mean overall survival of patients with MPMN to be 97.2±15.0 months (3). The longest overall survival of a patient with MPMN documented to date is 288 months (39). The patient in the present case survived for >14 years and the increased survival time may be associated with the multidisciplinary treatment the patient received.

The findings of the present study have the following clinical implications. Firstly, if pre-operative biopsy is not performed, a correct diagnosis may not be obtained, thus delaying treatment for MPMNs. A more standardized diagnosis procedure, including pre-operative biopsy, would be required in future for an improved outcome. Secondly, with the exception of recurrence of primary lesions or metastases, the occurrence of novel MPMNs should be considered during follow-up. Thirdly, multidisciplinary treatment can substantially increase the survival of patients with MPMNs.

In the present study, multidisciplinary treatment based on surgery exhibited a significant survival advantage for a patient with a rare case of MPMN. However, further research with additional cases, particularly including patients with more than two MPMNs, is required to definitively prove the clinical benefit of multidisciplinary treatment for the overall survival of patients with MPMNs.

## Figures and Tables

**Figure 5:**
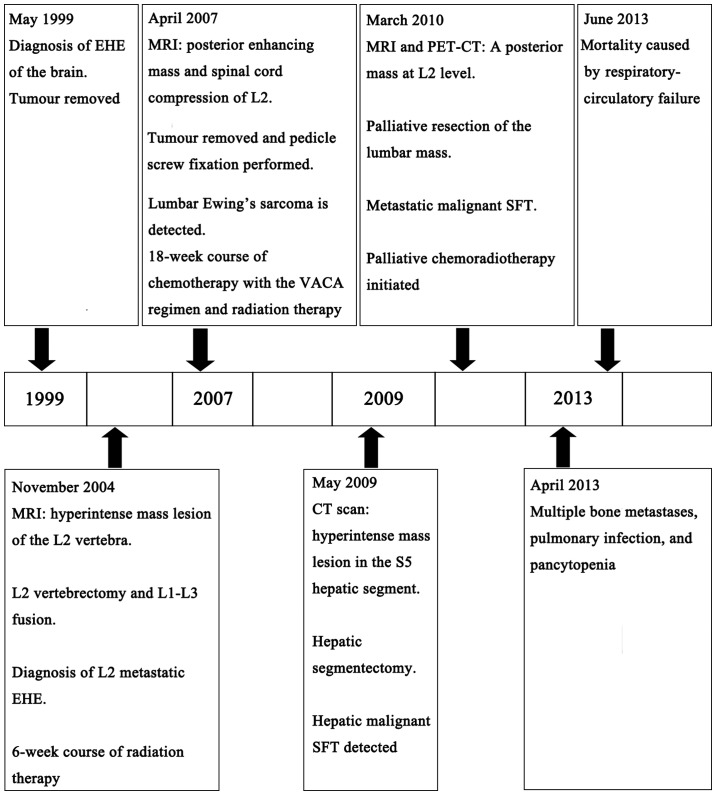
Summary of the most significant procedures and diagnoses since a 45-year-old woman received an initial diagnosis of EHE of the brain. The patient eventually succumbed to respiratory-circulatory failure following multiple bone metastases, lung infection and pancytopenia. EHE, epithelioid hemangioendothelioma; MRI, magnetic resonance imaging; VACA, vincristine, dactinomycin, cyclophosphamide and adriamycin; PET-CT, positron emission tomography-computed tomography; SFT, solitary fibrous tumour; L1-3, lumbar 1-3.
